# Brainstem dysfunction in critically ill patients

**DOI:** 10.1186/s13054-019-2718-9

**Published:** 2020-01-06

**Authors:** Sarah Benghanem, Aurélien Mazeraud, Eric Azabou, Vibol Chhor, Cassia Righy Shinotsuka, Jan Claassen, Benjamin Rohaut, Tarek Sharshar

**Affiliations:** 1 Department of Neurology, Neuro-ICU, Sorbonne University, APHP Pitié-Salpêtrière Hospital, Paris, France; 2 Medical ICU, Cochin Hospital, AP-HP, Paris, France; 3 Department of Neuro-ICU, GHU-Paris, Paris-Descartes University, Paris, France; 4 Laboratory of Experimental Neuropathology, Pastuer Institute, Paris, France; 5 Department of Physiology, Clinical Neurophysiology Unit, APHP, Raymond Poincaré Hospital, University of Versailles Saint Quentin en Yvelines, Garches, France; 6 Department of Intensive Care Medicine, Saint-Joseph Hospital, Paris, France; 7 Intensive Care Unit and Postgraduate Program, Instituto Nacional de Câncer, Rio de Janeiro, Brazil; 8 D’Or Institute for Research and Education, Rio de Janeiro, Rio de Janeiro Brazil; 9 Department of Neurology, Neuro-ICU, Columbia University, New York, NY USA; 10 Institut du Cerveau et de la Moelle épinière, ICM, INSERM UMRS 1127, CNRS UMR 7225, Pitié- Salpêtrière Hospital, Paris, F-75013 France

**Keywords:** Brainstem dysfunction, Brain injured patients, Intensive care unit, Sedation, Brainstem reflexes, Disorders of consciousness, Autonomic nervous system, Neurological respiratory failure, Immune reflex, Auditory and somatosensory evoked potentials and electroencephalogram

## Abstract

The brainstem conveys sensory and motor inputs between the spinal cord and the brain, and contains nuclei of the cranial nerves. It controls the sleep-wake cycle and vital functions via the ascending reticular activating system and the autonomic nuclei, respectively. Brainstem dysfunction may lead to sensory and motor deficits, cranial nerve palsies, impairment of consciousness, dysautonomia, and respiratory failure. The brainstem is prone to various primary and secondary insults, resulting in acute or chronic dysfunction. Of particular importance for characterizing brainstem dysfunction and identifying the underlying etiology are a detailed clinical examination, MRI, neurophysiologic tests such as brainstem auditory evoked potentials, and an analysis of the cerebrospinal fluid. Detection of brainstem dysfunction is challenging but of utmost importance in comatose and deeply sedated patients both to guide therapy and to support outcome prediction. In the present review, we summarize the neuroanatomy, clinical syndromes, and diagnostic techniques of critical illness-associated brainstem dysfunction for the critical care setting.

## Introduction: the concept of brainstem dysfunction

The brainstem is the caudal portion of the brain that connects the diencephalon to the spinal cord and the cerebellum [1]. The brainstem mediates sensory and motor pathways between the spinal cord and the brain and contains nuclei of the cranial nerves, the ascending reticular activating system (ARAS), and the autonomic nuclei. It controls the brainstem reflexes and the sleep-wake cycle and is responsible for the autonomic control of the cardiocirculatory, respiratory, digestive, and immune systems. Brainstem dysfunction may result from various acute or chronic insults, including stroke, infectious, tumors, inflammatory, and neurodegenerative diseases. In the context of critical illness, the brainstem can be susceptible to various insults that can be categorized as structural and non-structural origin. Brainstem dysfunction can then contribute to impairment of consciousness, cardiocirculatory and respiratory failure, and thus increased mortality [2–5].

In the present review, we describe brainstem functional neuroanatomy, clinical syndromes, and assessment methods before addressing the concept of critical illness-associated brainstem dysfunction.

## Functional neuroanatomy of the brainstem

**Fig. 1:**
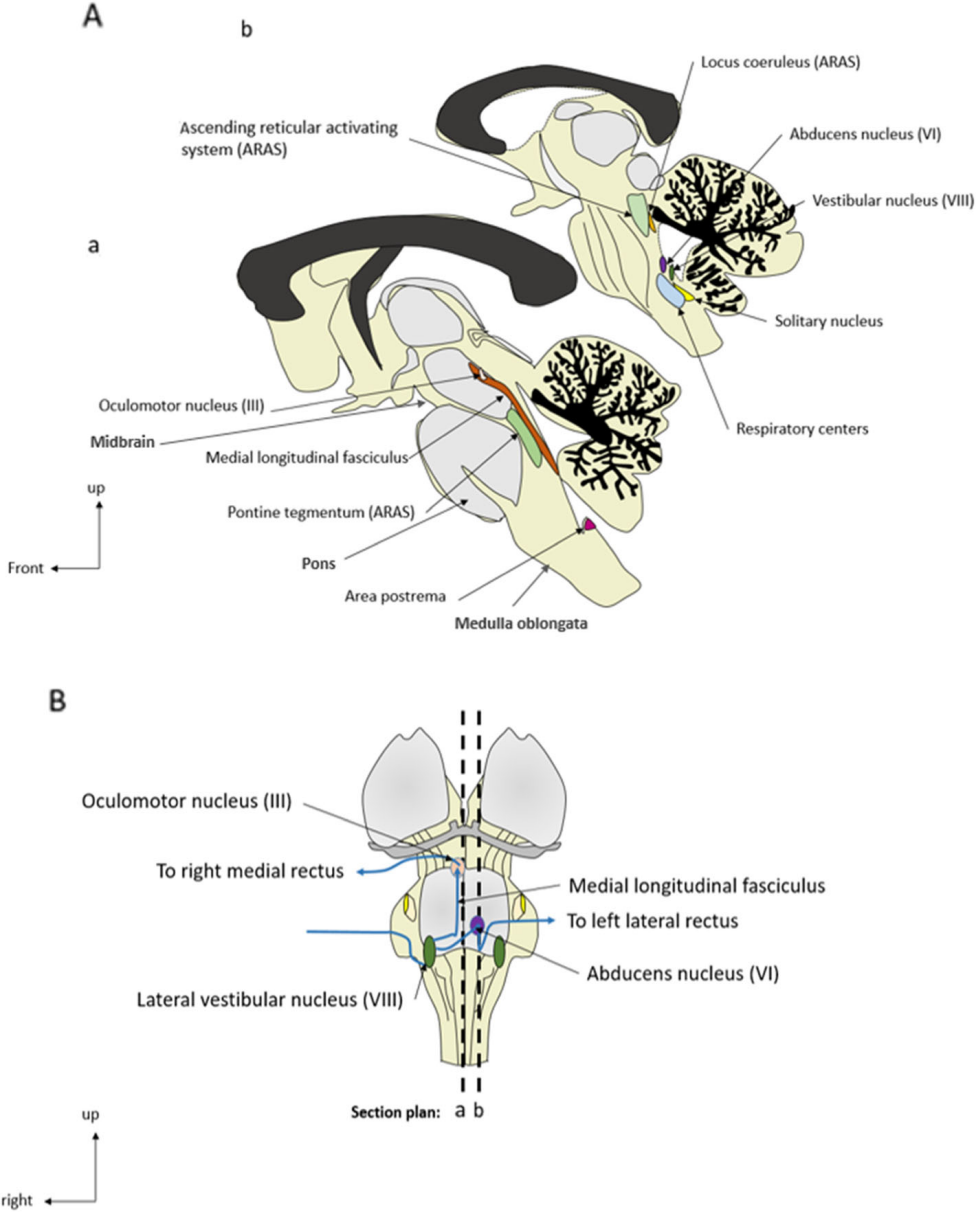
General anatomy of the brainstem and oculocephalic circuit. **A** Anatomical sagittal sections. **B** Representation of the sagittal section plans and of the oculocephalic circuit (ventral part)

**Fig. 2:**
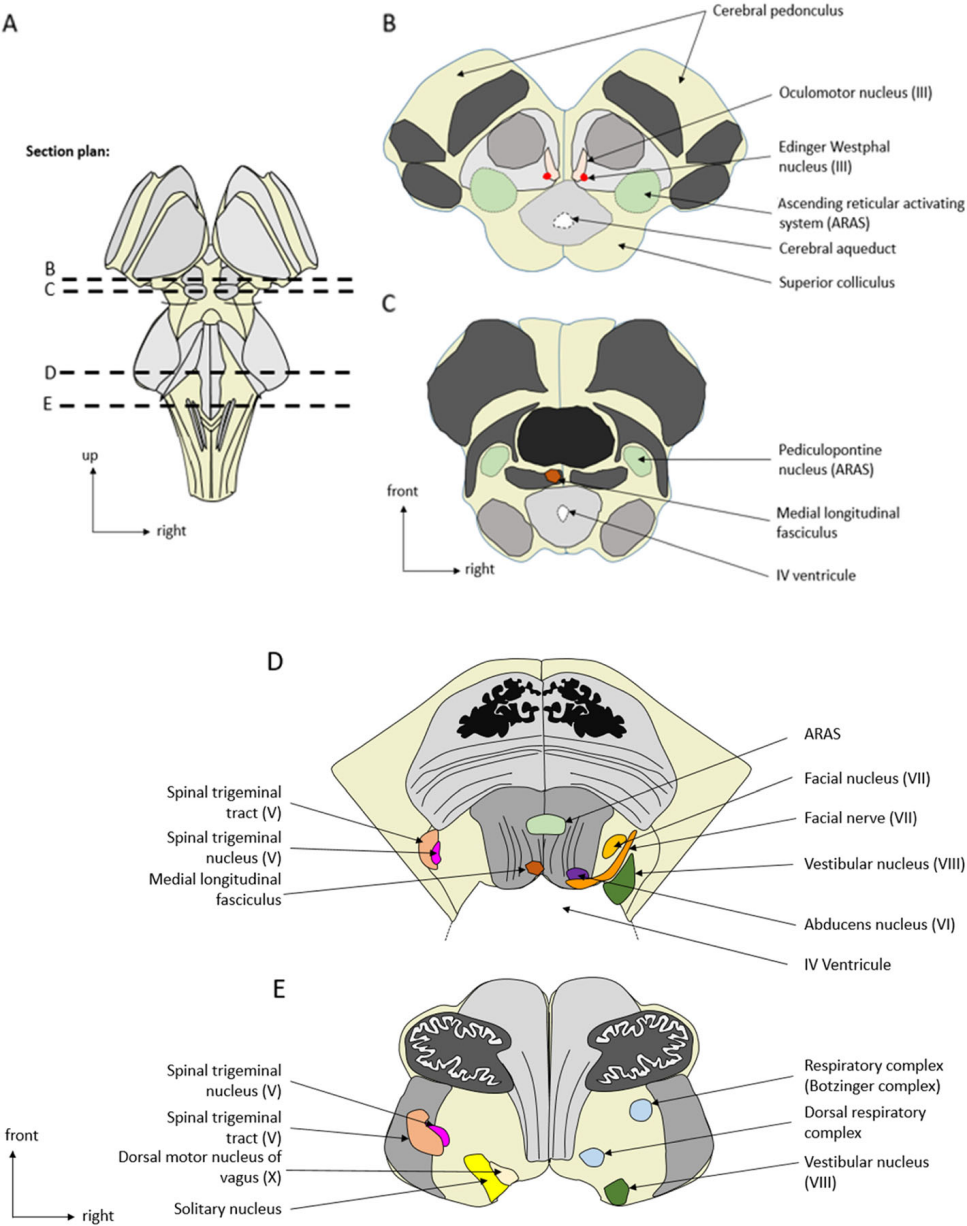
Anatomical axial sections of the brainstem. **a** Representation of the brainstem (dorsal part) and of the axial sections plans. **b**, **c** Midbrain axial sections. **d** Pons axial sections. **e** Medulla oblongata axial sections

The brainstem can be categorized into three major parts: midbrain, pons, and medulla oblongata (Figs. 1 and 2). The brainstem contains both gray and white matter, with the basilar artery representing the vascular supply. The gray matter includes the nuclei of the cranial nerves (anterior part), the ARAS (posterior part), the extrapyramidal and the central autonomic nervous system (ANS). This gray matter controls brainstem reflexes, arousal, automatic movements, and homeostasis, respectively. The white matter is composed of ascending sensory pathways and descending pyramidal and extrapyramidal pathways (Table 1).

**Table 1:** Functional neuroanatomy for the intensivist

	Anatomic structures	Function
Gray matter	Nuclei of cranial nerves	Brainstem reflexes
	Nuclei of ascending reticular activating system (ARAS)	Arousal, sleep/wake cycles, and alertness
	Nuclei of the extrapyramidal system	Automatic movements
	Nuclei of the central autonomic system	Vital function regulation and homeostasis
White matter	Axons of ascending pathways: Posterior column-medial lemniscus pathway Spinothalamic tract and lateral lemniscus pathway	Sensory information: Fine touch, vibration, two-point discrimination, and proprioception Pain and temperature
	Axons of descending pathways: Pyramidal corticospinal and corticobulbar tracts Extrapyramidal tract (rubrospinal, pontine and medullary reticulospinal tract, lateral vestibulospinal and tectospinal tracts)	Voluntary motor control Reflexes, locomotion, complex movements, and postural control

## Brainstem syndromes and assessment

Brainstem pathology should be considered in cases of (a) sensory or motor deficits combined with cranial nerve palsy, (b) impairment of consciousness, (c) dysautonomia, or (d) neurological respiratory failure.

### Brainstem motor and sensory deficits and cranial nerve palsy

The pyramidal and extrapyramidal tracts connect the upper motor neurons and the extrapyramidal nuclei with the lower motor neurons located in either the brainstem or the spinal cord [6]. While the former controls voluntary movement, the latter is involved in reflexes, motion, complex movements, and postural control (Tables 1 and 2). Upper motor neuron damage can lead to symptoms, ranging from hemiparesis to the locked-in syndrome, which is typically characterized by intact awareness, quadriplegia, anarthria, and absence of eye movements except for preserved vertical gaze. It usually results from bilateral pontine white matter lesions [7]. Characteristic clinical features of brainstem lesions include ipsilateral cranial nerve palsies or cerebellar signs combined with contralateral motor deficits. Brainstem lesions may present with abnormal movements, such as hemichorea, hemiballism, dystonia, tremor, asterixis, pseudo-athetosis, and non-epileptic myoclonus [8] (Table 2). Bilateral motor corticobulbar tract lesion may present with swallowing impairment, dysphagia, dysphonia, velo-pharyngo-laryngeal impairments, uncontrollable crying/laughing episodes, and emotional lability (i.e., pseudobulbar affect; Table 2). A brainstem lesion of the posterior column-medial lemniscus pathway and the spinothalamic tract results in a contralateral proprioceptive/touch and temperature/pain deficit, respectively.

**Table 2:** Functional anatomy of the brainstem

Brainstem structures	Functions	Centers	Symptoms
Midbrain (rostral to the pons and caudal to the thalamus and the basal ganglia)	Eye movements	Cranial nerve nuclei: III oculomotor nerve (mainly motor) IV trochlear nerve (motor)	Oculomotor signs: Ptosis (III) Ophthalmoplegia (III, IV)
	Pupillary size: sphincter pupillae and muscles of the ciliary body, pupil light reflex	Cranial nerve nuclei: III oculomotor nerve	Pupillary anomalies: Myosis (sympathetic lesion) Mydriasis (parasympathetic lesion) Anisocoria
	Movement control	Substantia nigra	Parkinsonian syndrome and movement disorders (hemichorea, hemiballism, dystonia, tremor, asterixis, pseudo-athetosis, non-epileptic myoclonus)
	Posture tone	Red nucleus	Postural tone impairment
	Posture/auditory and visual integration	Accessory optic tractus	Balance disorder
	Posture and movement integration	Tectum (dorsal part)	Balance disorder
	Posture and inhibitor motor centers	Tegmentum (ventral portion) (basal ganglia and thalamus connections)	Involuntary movements
	Sleep/wake cycles, alertness, and arousal	ARAS: composed of almost 100 nuclei, including locus coeruleus-raphe nuclei with neocortex connections	Sleep disturbance Consciousness disorders
	Central thermic regulation	ARAS-hypothalamus connections	Hypo/hyperthermia
Pons (between the medulla and the midbrain)	Facial sensitivity, muscles of mastication	Cranial nerve nuclei: V trigeminal nerve (sensory and motor)	Facial symptoms: Facial dysesthesia Oculomotor signs: Corneal/ciliary reflex impairment
	Facial muscles and taste from the anterior 2/3 of the tongue (VII)	Cranial nerve nuclei: VII facial nerve (sensory and motor)	Facial symptoms: Peripheral facial palsy
	Eye movement (abduction)	Cranial nerve nuclei: VI abducens nerve (motor)	Oculomotor signs: Ophthalmoplegia
	Posture, sensation of rotation, gravity, and sound	Cranial nerve nuclei: VIII vestibulocochlear nerve (mostly sensory) Cerebellum tract	Altered audition (VIII) Balance disorders (VIII and cerebellum tract)
	Posture Posture and inhibitor motor center	Spinocerebellar tracts Tegmentum (thalamus and basal nuclei connections)	Cerebellar ataxia Involuntary movement
	Motor efference integration Sensory efference integration	Tracts carrying signals to the thalamus	Motor deficit Sensory deficit
	Consciousness, alertness, and sleep regulation	Tracts carrying signals to the thalamus	Sleep disturbance Consciousness disorders
	Sleep/wake cycles, alertness, and arousal	ARAS: composed of almost 100 nuclei, including raphe nuclei and locus coeruleus-raphe nuclei-neocortex connections	Sleep disturbance Consciousness disorders
	Emotion	ARAS: locus coeruleus and amygdala connections	Anxiety and post-traumatic stress disorder (PTSD)
	Central thermic regulation	ARAS-hypothalamus connections	Hypo/hyperthermia
	Respiratory drive: respiratory rate and tidal volume control	Pedunculopontine tegmentum, locus coeruleus, lateral parabrachial respiratory group, and Kölliker-Fuse nuclei	Respiratory drive dysfunction: Kölliker-Fuse and parabrachial nuclear: increase tidal volume, decrease respiratory rate Lower part/ponto-peduncular injury: respiratory asynchronism
Medulla (lower half of the brainstem, connects the higher levels of the brain to the spinal cord)	Taste from the posterior 1/3 of the tongue	Cranial nerve nuclei: IX glossopharyngeal (sensory and motor)	Tongue sensory impairment
	Pharyngo-laryngeal reflex	Cranial nerve nuclei: IX glossopharyngeal nerve X vagus nerve (sensory and motor) XI spinal nerve (motor)	Oro-pharyngo-laryngeal anomalies: Dysphagia (swallowing impairment) Dysphonia Velo-pharyngo-laryngeal impairment Absence of pharyngeal/gag reflex
	Glossal muscles	XII hypoglossal (mainly motor)	Tongue motor impairment (fasciculation, motor deficit)
	Cough	IX glossopharyngeal nerve X vagus nerve	Absence of cough reflex (IX, X)
	Posture	Spinocerebellar tracts	Cerebellar ataxia
	Regulation of autonomic nervous system:	Sympathetic nuclei Parasympathetic nuclei: vagus nerve (X) control of the heart, lung, digestive tracts	Autonomic dysfunction
	Cardiac regulation	Sympathetic nuclei Parasympathetic nuclei: vagus nerve (X) control of the heart, lung, digestive tracts	Oculocardiac reflex impairment (X) Dysautonomia: tachycardia (parasympathetic impairment), bradycardia (sympathetic impairment), sudden death
	Vasomotor regulation		Hemodynamic failure: Dysautonomia with hypertension (parasympathetic impairment), hypotension (sympathetic impairment)
	Gastrointestinal motility		Gastrointestinal motility anomalies
	Respiratory drive: respiratory rate and tidal volume control	Respiratory centers: dorsal respiratory complex	Respiratory drive dysfunction: respiratory rate irregularities and ataxic breathing, hyperventilation, respiratory-ventilator asynchronism, central apnea
	Microbiota gut-brain axis, senses and peripheral inflammation modulation	Vagus nerve (X)	Maladaptive immune response, gut-brain axis impairment
Tracts all along the brainstem	Connection of the oculomotor nerves (see Fig. 1)	Medial longitudinal fasciculus	Internuclear ophthalmoplegia
	Motor information from the periphery to supratentorial structures	Corticospinal tract Pyramidal and extrapyramidal tracts	Motor deficit, locked-in syndrome Tetrapyramidal and extrapyramidal syndromes with movement disorders (tremor) Non-epileptic myoclonus
	Sensory information from the periphery to supratentorial structures	Posterior column-medial lemniscus pathway and spinothalamic tracts	Sensory deficit
	Oculosympathetic control	Centers control of the ciliary nerve, superior tarsal muscle, pupillary sphincter/dilator	Horner’s syndrome (ptosis, myosis, enophtalamos, anhidrosis)

The testing of the cranial nerves and brainstem reflexes is described in Table 3. Abnormal spontaneous eye position and movements may be encountered in patients with brainstem lesions and can be seen in comatose patients. Assessment of pupillary size allows the diagnosis of third nerve lesion (i.e., mydriasis) or Horner’s syndrome (i.e., myosis, ptosis, enophtalmia, and anhidrosis). Pupillary light, corneal, oculocephalic, and gag reflexes are routinely assessed in the critical care setting. The oculovestibular responses and oculocardiac are less frequently tested, except to determine brain death. The absence of brainstem reflexes and spontaneous breathing is a prerequisite for the diagnosis of brain death [9]. Automated pupillometry could improve the assessment of the pupil light reflex and thereby its prognostic value [10]. Corneo-mandibular reflexes can be detected in acute brain injury, but its prognostic relevance remains controversial. Finally, assessments of primitive reflexes are less relevant in the ICU context but can be seen in patients with neurodegenerative disease (Table 3).

**Table 3:** Brainstem reflexes neuroanatomical and clinical description

Reflex	Examination technique	Normal response	Afferent pathway	Brainstem centers	Efferent pathway
Physiological reflexes					
Pupillary light reflex	Response to light	Direct and consensual myosis followed by mydriasis	Retina, optic nerve, chiasma, optic tract	Pupillo-constrictor: midbrain, pretectal olivary nucleus, Edinger-Westphall nucleus Pupillo-dilator: posterior-lateral hypothalamus, cervical ganglion, trigeminal ganglion, abducens	Sympathetic fibers of cranial nerve III (oculomotor)
Cilio-spinal	Latero-cervical nociceptive stimulation	Uni- or bilateral irido-dilatation	Sensory ascending pathways to centro-spinal centers	Midbrain	Cranial nerve III
Fronto-orbicular	Glabellar percussion	Eyes closing	Cranial nerve V (trigeminal)	Pons	Cranial nerve VII (facial)
Oculocephalic	Turn head from side to side	Eyes move conjugately in direction opposite to head	Semicircular canals, Cranial nerve VIII (oculovestibular)	Pons, nucleus vestibularus, nucleus abducens	Cranial nerves III (oculomotor) and VI (abducens)
Oculovestibular	Irrigate external auditory canal with 50 ml of cold water	Nystagmus			
Corneal	Stimulation of cornea with saline drops	Eyelid closure	Cranial nerve V (trigeminal)	Pons, trigeminal and facial nuclei	Cranial nerve VII (facial)
Grimace/masseterian	Deep pressure on nail bed, supraorbital ridge, or temporo-mandibular joint	Facial grimace and limb movement			
Cough reflex	Stimulation of the carina with a suction tube	Cough	Cranial nerve IX (Glossopharyngeal) and X (vagal)	Medulla, nucleus tractus solitarius	Cranial nerve IX (glossopharyngeal) and X (vagal)
Gag/pharyngeal reflex	Stimulation of the soft palate	Symmetrical rise of soft palate gag reflex			
Oculocardiac	Ocular globe compression	Decrease in heart rate	Cranial nerve V (trigeminal)	Pons, medulla	Cranial nerve X (vagal)
Primitive reflexes					
Palmo-mental	Pressure of the thenar eminence with a thin stick	Single twitch of the ipsilateral mentalis muscle	Posterior column-medial lemniscus pathway	Pons	Cranial nerve VII (facial)
Corneo-mandibular	Corneal stimulation	Contralateral deviation of the jaw	Cranial nerve V (trigeminal)	Pons	Cranial nerve VII (facial)
Other syndromes					
Internuclear ophthalmoplegia (see Fig. 1)	Oculomotricity testing Can be observed during oculocephalogyric or oculovestibular tests	Disconjugate lateral gaze with a preserved convergence	Lesion of the medial longitudinal fasciculus	Connects the sixth nucleus with the contralateral third nucleus	
Claude Bernard-Horner’s syndrome	Ptosis, myosis, enophtalamos, anhidrosis		Sympathetic pathway injury		
Vertical nystagmus, skew deviation			Midbrain or medulla injury		
Ocular bobbing			Pons injury		

When suspecting brainstem lesions, MRI will have the highest yield to further localize and characterize brainstem lesions [6] (Table 4). Evoked potentials may be also useful for detecting a brainstem lesion. EEG [11] may be supportive in patients with abnormal movements and disorders of consciousness, and cerebrospinal fluid (CSF) analysis for those with suspected inflammatory or infectious diseases.

**Table 4:** Acute and chronic diseases involving the brainstem

Causes of brainstem dysfunction	
Acute primary insult	
Vascular injury	
Ischemic: thrombotic or cardio-embolic, lacunar ischemia due to small vessel disease, vasculitis	
Hemorrhage	
Inflammatory	
Multiple sclerosis (MS)	
Acute disseminated encephalomyelitis (ADEM)	
Neuromyelitis optica (NMO) (anti-MOG, anti-AQP4 antibodies, or seronegative types)	
Birkenstaff encephalitis (anti-ganglioside GQ1b antibodies)	
Behcet disease and rarely other autoimmune disease (lupus, neuro-sarcoidosis)	
Langerhans cell histiocytosis	
Traumatic: direct or indirect injury	
Metabolic: central pontine myelinolysis	
Infectious: rhombencephalitis, abscess, *Listeria monocytogenes* and enterovirus 68 and 71, followed by herpes simplex viruses and tuberculosis, Epstein-Barr virus (EBV), and human herpesvirus 6 (HHV6)	
Paraneoplastic (anti-neuronal NMDA, AMPA, GABA, CASPR2, Hu, Ma2, Ri, Yo, CV2, amphiphysin, Lgi1,glycine, mGluR1/5, VGKC/VGCC, GAD antibodies)	
Chronic primary insult	
Tumoural	
Degenerative/atrophic injury	

*MRI* magnetic resonance imaging, *TDM* tomodensitometry, *CSF* cerebrospinal fluid, *ECG* electrocardiogram

MRI results according to etiologies:

Vascular injury: diffusion and FLAIR-weighted sequence hyperintensity restricted to a vascular territory

Hemorrhage: SWI/T2* sequence hypointensity

Inflammatory: diffuse or multifocal white matter lesions on T2- and FLAIR-weighted sequences, with or without contrast enhancement

Inflammatory NMO (MRI of optical nerve and medullary MRI): extensive and confluent myelitis on more than three vertebrae and optical neuritis with possible contrast enhancement

Traumatic injury: hyperintensity on diffusion sequence, diffuse axonal injuries on DTI (diffusion tensor imaging) sequence, hemorrhage lesions on T2*/SWI

Metabolic: T2 hyperintensity specifically involves the central pons

Infectious: abscess/nodes with contrast enhancement

Paraneoplastic: limbic encephalitis with temporal diffusion and FLAIR hyperintensity

Tumor: mass with possible necrosis, contrast enhancement and oedema revealed by a FLAIR hyperintensity around tumor

Degenerative injury: brain and brainstem atrophy (colibri sign)

### Impairment of consciousness

The ARAS controls the sleep-wake cycle and includes several nuclei mainly located in the pontine and midbrain tegmentum [12] (Table 2, Figs. 1 and 2): the rostral raphe complex, the parabrachial nucleus, the laterodorsal tegmental nucleus, the locus coeruleus (LC), the nucleus pontis oralis, the basal forebrain, and the thalamus. Monoaminergic neurons are directly linked to the cortex and are inhibited during deep sleep. Cholinergic pedunculopontine and laterodorsal tegmental nuclei are indirectly connected to the cortex via the thalamus and remain active during rapid eye movement sleep. These pathways are modulated by hypothalamic neurons [13].

Disorders of consciousness can be organized between acute and subacute or chronic [14]. Acute impairments of consciousness include coma which is defined as a “state of unresponsiveness in which the patient lies with eyes closed and cannot be aroused to respond appropriately to stimuli even with vigorous stimulation” [14]. The association of a prolonged non-responsive coma with a complete cessation of brainstem reflexes and functions suggests the diagnosis of brain death which is defined as an irreversible loss of all functions of the entire brain. Delirium is defined as an acute and fluctuating disturbance of consciousness, including attention and impairment of cognition, associated with motor hyperactivity or hypoactivity [15, 16]. Delirium has been associated with long-term cognitive impairment, functional disability in ICU survivors, and hospital mortality [15]. Brainstem dysfunction could account for some features of delirium, such as fluctuations in arousal and attentional impairment that could be related to ARAS and to ponto-mesencephalic tegmentum dysfunction, respectively. Other states of acute impairment of consciousness include clouding of consciousness and stupor, but they are less frequently used [14].

Subacute or chronic disorders of consciousness include the vegetative state (VS, also called Unresponsive Wakefulness Syndrome) defined as state of unresponsiveness in which the patient shows spontaneous eye opening without any behavioral evidence of self or environmental awareness [17]. The minimally conscious state (MCS) is defined as state of severely impaired consciousness with minimal behavioral evidence of self or environmental awareness, characterized by the presence of non-reflexive behavior (visual pursuit, appropriate motor response to painful stimulus) or even intermittent command following indicating a cortical integration [18, 19]. The VS and MCS are related to a preservation of brainstem arousal functions but with persistent impairment of supratentorial networks implicated in consciousness [20]. Stimulation of the ARAS may improve consciousness in vegetative or MCS patients [21]. In addition to deep brain stimulation, vagal nerve stimulation, which probably modulates the activity of the nucleus of the tractus solitarius and the dorsal raphe, has shown promising results [22].

In addition to these classical syndromes, other consciousness impairments have been described. Peduncular lesions can cause hallucinations [23] which may be encountered in ICU patients. More generally speaking, it is likely that brainstem dysfunctions account for a portion of the sleep-wake cycle impairments experienced by ICU patients. Brainstem lesions can induce cognitive deficits including impaired attention, naming ability, executive functioning, and memory impairment [24], ascribed to a disruption of interconnection between the frontal-subcortical system and the brainstem [1]. Finally, deep sedation is a pharmacologically induced coma, and its mechanisms of action involve the brainstem GABA and *N*-methyl-d-aspartate (NMDA) receptors [25].

Assessments of consciousness are based on neurological examination to confirm the diagnosis, determine the underlying cause, and evaluate the prognosis. In clinical practice, this assessment most commonly relies on the Glasgow Coma Scale (GCS) [20]. Focusing on the brainstem in particular, the FOUR (Full Outline of UnResponsiveness) score is to be preferred as it includes the corneal, pupil light, and cough reflexes and respiratory patterns [26]. In comatose patients, pupil sizes and reactivity can be suggestive of particular etiologies, such as drug overdose (myosis for opioids or mydriasis for tricyclic anti-depressants). In comatose brain-injured patients, brainstem reflex assessment is crucial to detect a uncal or downward cerebellar (tonsillar) herniation [10]. While the absence of corneal and pupillary light reflexes is strongly associated with poor outcome in post-anoxia, their prognostic value is less validated in other causes [27].

Patients with severe critical illness may be comatose due to sedation, which in clinical practice can be assessed using the RASS (Richmond Agitation Sedation Scale) [28]. In deeply sedated patients (i.e., RASS − 4 or − 5), the Brainstem Reflexes Assessment Sedation Scale (BRASS) might be useful to assess the effect of sedatives on the brainstem and potentially detect a brainstem dysfunction [29] (Table 5). The CAM-ICU and ICDSC are appropriate to monitor delirium [16, 30]. Finally, in VS and MCS patients, the Coma Recovery Scale-Revised has also been validated [20].

**Table 5:** Brainstem Reflexes Assessment Sedation Scale (BRASS)

Variable	Score point
Absence of cough reflex	1
Absence of pupillary light reflex	1
Absence of corneal reflex	2
Absence of grimacing to pain and absence of OCR	1
Absence of grimacing to pain and presence of OCR	3

*OCR*: oculocephalic reflex

BRASS is a clinical score that has been developed for scoring brainstem dysfunction in deeply sedated, non-brain-injured, mechanically ventilated, critically ill patients and ranges from 0 to 7

The BRASS has prognostic value, as 28-day mortality proportionally increases with the BRASS score

Coma due to structural brainstem lesions is predominantly related to pedunculo-pontine tegmental lesions, usually detected on MRI [12] (Table 4). Neurophysiological tests may be useful to assess the neurological prognosis in patients with impairment of consciousness. Somatosensory evoked potentials (SSEP) assess conduction from peripheral nerves (N9) to the somatosensory cortical (N20) regions passing through the brainstem (P14). Brainstem auditory evoked potentials (BAEP) are described in Table 6 [11]. Interestingly, sedation increases latencies and decreases amplitudes of evoked potentials in a dose-dependent manner but does probably not change the amplitudes with low to moderate doses used in ICU [31].

**Table 6:** BAEP waves and blink test

BAEP waves	Anatomic localization
I	Distal portion of the auditory nerve
II	Proximal portion of the auditory nerve or cochlear nuclear complex, in the upper part of the medulla, ipsilateral to the stimulation side
III	Cochlear nucleus or superior olivary complex in caudal pontine tegmentum, ipsilateral to the stimulation side
IV	Superior olivary complex (lateral lemniscus), contralateral to the stimulation side
V	Inferior colliculus located in the midbrain, contralateral to the stimulation side
Blink test	Response
After stimulation of the supraorbital nerve, three responses are recorded on eyelid orbicular muscles: an early ipsilateral (R1) response and the two (ipsi- and contralateral) late responses (R2)	R1 response generated at the level of the pons, R2 responses at the level of the trigeminal-spinal tract at the pons level, the medulla oblongata, and the caudal trigeminal-spinal nucleus

Brainstem lesions can result in absent or delayed peaks III and V, prolonged III–V and I–V inter-peak latency, or a reduced I/V amplitude ratio (< 0.5)

Delay or absence of R1 indicates a facial /trigeminal nerve injury. R2 can be delayed in comatose patient and is also bilaterally delayed or absent in Wallenberg’s syndrome (with a R1 preserved)

The intracranial conduction time and intrapontine conduction time are assessed by measures of the P14–N20 inter-peak latency on SSEP and the III–V inter-peak latency on BAEP [11]. The prognostic value of BAEP has been explored in various causes of coma [32–34]. After cardiac arrest, the predictive value of BAEP for poor outcomes is limited [35]. However, in traumatic brain injury, preserved BAEP are associated with a good outcome [36]. Wave I can disappear if the auditory nerve is injured (traumatic or hypoxic injuries) [37].

Reactivity on EEG to auditory, visual, or nociceptive stimuli is important to assess after cardiac arrest because its absence is associated with poor outcome [38, 39]. Absent reactivity can result from a thalamus-brainstem loops and ARAS dysfunction [40–43]. The electrophysiological measurement of the blink reflex (Table 6) is a way to study the trigemino-facial loop [44], but its prognostic value in comatose patients remains insufficiently supported [45].

### Autonomic nervous system impairment

The ANS plays a key role in homeostasis and allostasis by controlling vital functions and the immune system [46] and is composed of sympathetic (e.g., noradrenergic) and parasympathetic (e.g., cholinergic) systems. Sympathetic effects originate from the spinal cord (D1 to L3), while parasympathetic neuronal cell bodies are present in the nuclei of cranial nerves III (Edinger Westphal nuclei), VII, IX, and X and the sacral spinal cord (S2 to S4). Activation of the parasympathetic nervous system results in a decrease in heart rate (HR) and blood pressure (BP), and an increase in gastrointestinal tonus, vesical detrusor contraction, and myosis. Activation of the sympathetic system results in opposite effects. Cortical input can modulate responses in the ANS [46] as well as various receptors throughout the body, including the baroreceptors [47].

Brainstem injury may cause dysautonomic symptoms, which can be life-threatening [48] (Table 2). Cardiac arrhythmias frequently occur after brainstem stroke and are associated with increased mortality [48]. An intracranial hypertension-induced midbrain insult can impair parasympathetic control and thereby induce adrenergic storm. In brain death, there is a disappearance of the vasomotor tone and an impairment of myocardial contractility [49]. As exhaustive discussions of tests that allow testing of the ANS are beyond the scope of this review, we will focus on cardiovascular tests applicable to ICU patients. Standard monitoring allows for the detection of variations in HR and BP that can be suggestive of dysautonomia. However, the lack of apparent changes in cardiovascular signals does not rule out dysautonomia, which can be then assessed with the HR and BP spectral analysis. High frequency (HF) band (i.e., 0.15 to 0.4 Hz) variability of the HR is thought to predominantly reflect parasympathetic tone, while low frequency (LF) variability (i.e., 0.04 to 0.15 Hz) is primarily mediated by sympathetic activity. The LF/HF ratio reflects the sympathovagal balance. Therefore, spectral analysis allows studying the sympathetic, parasympathetic, and baroreflex activities both at rest and during stimulation [50]. If the Valsalva maneuver, the cold pressure test, and the pharmacological tests (with yohimbine or clonidine) allow testing the ANS, their use in ICU is very limited. Conversely, pupillometry is much more applicable for assessing dysautonomia in ICU. Thus, patients with dysautonomia present a pupil dilatation at resting state and a slow redilatation time [51].

### Neurogenic respiratory failure

There are two types of muscles that play a major role in the respiratory system, dilatator muscles of the superior airway that are innervated by the brainstem via cranial nerves (motor neurons present in the V, VII, and XII nuclei) and contractor/pump muscles (diaphragm, intercostal, sternocleidomastoid, abdominal muscles) that are innervated by spinal motor neurons. They are controlled by bulbospinal (automatic command) and corticospinal (voluntary command) pathways. The respiratory drive originates from neurons of the latero-rostro-ventral medulla oblongata, which includes the pre-Botzinger complex and the parafacial respiratory group that control inspiration and expiration, respectively [52] (Table 2). This center receives various inputs to automatically adjust the respiratory drive to metabolic and mechanic changes [53]. Metabolic inputs are mediated by both peripheral (aortic and carotid) and central (medulla oblongata and LC) chemoreceptors [54]. The mechanical inputs are mediated by mechanoreceptors localized in the pulmonary parenchyma, bronchial wall, and muscle. At the level of the pons, the pedunculopontine tegmentum, the LC, the lateral parabrachial and Kölliker-Fuse nuclei are involved in the automatic respiratory control [55] (Table 2).

Automatic and voluntary control of respiratory motor neurons can be injured together or separately. For instance, automatic control is impaired in central congenital and acquired hypoventilation syndrome (i.e., Ondine syndrome), while voluntary control is preserved [56]. Acquired hypoventilation syndrome can result from brainstem tumoral, traumatic, ischemic, and inflammatory injuries [57], which implies the need for long-term mechanical ventilation.

Ventilator management may be significantly affected by brainstem lesions, and importantly, clinical features of neurological respiratory dysfunction are related to the localization of brainstem injury. The more caudal the lesion is, the more it is associated with an impairment of the respiratory drive. Midbrain injuries do not usually affect the respiratory rate (RR). Injuries to the upper pons increase the tidal volume and decrease the RR, while injuries of the lower pons are associated with respiratory asynchrony (e.g., ponto-peduncular injury). Ataxic breathing (irregular pauses and apnea periods) and central apnea are observed in rostro-ventral medulla oblongata injuries and associated with poor outcomes. Central neurogenic hyperventilation results from activation of the medullary respiratory center. Finally, yawning or refractory hiccups may be seen with lesions of the posterolateral medulla oblongata [58]. Swallowing impairment contributes also to the difficulty of weaning mechanical ventilation and can be an indication for a tracheostomy.

There are various structural and non-structural causes of neurological respiratory dysfunction, including infratentorial lesions, drug toxicity, heart failure, and sepsis [59–61]. Diagnosis relies on standard assessments of respiratory function (e.g., ventilator curves, tidal volumes (Vt), and RR in mechanically ventilated patients) but also on assessing the ventilatory response to hypercapnia (e.g., during a t-piece trial). An electromyogram of the respiratory muscles, notably the diaphragm, provides relevant information on the central drive. This technique may be helpful in patients that are impossible to wean from mechanical ventilation. As a caveat, it may be at times difficult to differentiate central respiratory dysfunction from critical illness neuropathy/myopathy. EMG and nerve conduction studies may help with the distinction, but limited assessments of every respiratory muscle group and available at highly specialized units limit this approach [62]. In mechanically ventilated patients, spirography can be performed (with the Vt/inspiration duration (Ti) ratio reflects the ventilatory command intensity) as well as the occlusion pressure measurement (i.e., P0.1). The latter reflects the “unconscious”/central respiratory command, but variability of its measurements limits routine application.

## Brainstem dysfunction in critically ill patients

The leading causes of primary brainstem dysfunction are summarized in Table 4 and major differential diagnosis of brainstem dysfunction in Table 7. In the following section, we will discuss evidence for brainstem dysfunction encountered in critically ill patients beyond primary brainstem dysfunction.

**Table 7:** Differential diagnosis of brainstem dysfunction

Brainstem dysfunction	Differential diagnosis
Oculomotor anomalies (III, IV, VI cranial nerves nuclei)	Cranial nerve palsy Myopathy involving oculomotor muscles Neuromuscular disorders: myasthenia, Lambert-Eaton syndrome and botulism
Pupillary size anomalies	Anisocoria: compressive lesion of the III cranial nerve such as herniation/intracranial hypertension and posterior communicative artery aneurysm
	Mydriasis: third nerve lesion
Claude Bernard-Horner’s syndrome (ptosis, myosis, enophtalmia, anhidrosis)	Pancoast tumor Carotid or aortic dissection
Facial sensory anomalies (V cranial nerve nucleus)	Contralateral brain injury Cranial nerve palsy (V)
Facial motor anomalies (VII cranial nerve nucleus)	Contralateral brain injury Cranial nerve palsy (VII) Myopathy with facial paralysis Neuro-muscular disorders: myasthenia, Lambert-Eaton syndrome and botulism
Posture and movement anomalies	Uni- or bilateral basal ganglia lesions
Motor and/or sensory deficit	Contralateral brain injury Critical illness neuromyopathy Guillain-Barre syndrome
Motor deficit	Myopathy Neuro-muscular disorders: myasthenia, Lambert-Eaton syndrome and botulism
Autonomic (sympathetic and parasympathetic) dyfunctions	Spine injury Guillain-Barre syndrome
Respiratory control anomalies	Cervical spine injury (C3–C5) Phrenic nerve palsy Diaphragmatic injury Critical illness neuromyopathy Neuromuscular disorders: myasthenia, Lambert-Eaton syndrome and botulism

### Clinical features

The “brainstem dysfunction” hypothesis originates from our study on usefulness of neurological examination in non-brain-injured critically ill patients who required deep sedation. These patients have usually a severe critical illness and therefore a higher risk to develop severe secondary brain insult [3, 29]. Furthermore, protracted deep sedation is still required in more than 30% of critically ill patients [63] and has been reported to be associated with increased mortality [63]. We found that assessment of brainstem reflexes was reproducible in this population [3, 29]. We also found that routinely used sedative and analgesic agents such as midazolam and fentanyl do not impair pupillary light, corneal, and cough reflexes in 90% of cases but depress oculocephalic response and grimacing to painful stimulation (absent in 50 and 70%, respectively) [3, 29, 63]. The cessation of brainstem reflexes results from the combining effects of critical illness (i.e., secondary brain insult), sedative, and analgesic agents. It is interesting to note that Guedel observed more than 70 years ago that sedative drugs abolish brainstem reflexes according to a sequential pattern (the loss of consciousness, followed by the cessation of brainstem reflexes in a rostro-caudal way until apnea) [64].

In deeply sedated non-brain-injured critically ill patients, the cessation of brainstem responses follows two distinct patterns. The first is characterized by a depression of whole brainstem responses (similar to Guedel’s description), and the second is characterized by a preferential impairment of the corneal reflex, the pupillary light reflex, and to a lesser extent the cough reflex, with paradoxical preservation of the oculocephalic response. The latter profile is associated with the severity of critical illness and the depth of sedation. Interestingly, this pattern cannot be ascribed to a unique focal brainstem lesion which most likely relies on a functional rather than a structural origin. This suggests that some neuroanatomical centers are more sensitive to deep sedation, critical illness, or both. Opioids might also contribute to brainstem dysfunction, as they depress the ARAS, respiratory centers, and brainstem reflexes (notably pupillary light and cough reflexes). However, morphine infusion rates did not differ in our study between the two cessation patterns of brainstem reflexes [29].

To assess brainstem reactivity in deeply sedated critically ill patients, we developed the BRASS [29] (Table 5). The principle of the BRASS development is not in agreement with the traditional paradigm of Jackson, which states that the brainstem reflexes are abolished in a rostro-caudal way. It thus differs from the FOUR score [65], which conditions the assessment of the cough reflex to the cessation of the pupillary light and corneal reflexes. Besides improving the prediction of mortality in deeply sedated patients, the assessment of brainstem reflexes, with help of either the BRASS or the FOUR score, might prompt the ICU physician to perform a brainstem imaging. It is however likely that the processes involved in critical illness-related brainstem dysfunction are radiologically assessable.

### Electrophysiological, autonomic, and respiratory features of brainstem dysfunction

Neurophysiological tests provide further arguments for brainstem dysfunction in critically ill patients without primary brainstem injury. For instance, EEG is not reactive in 25% of patients with sepsis [42, 43], knowing that absence of reactivity can result from a dysfunction of the ARAS [40–43]. Middle latency BAEP responses and SSEP latencies were increased in 24% and 45% of deeply sedated non-brain-injured critically ill patients, respectively [34], indicating an impairment of the brainstem conduction. Interestingly, mean values of these latencies did not differ from those recorded in deeply sedated brain-injured patients.

Critical illness is also associated with decreased variability in HR and BP, with an impaired sympathetic tone and baroreflex [2, 50] and also with a reduced tidal volume variability [66] that can correlate with weaning failure. Since most of these findings concerned sedated patients, one may argue that sedative agents might be involved as a revealing or aggravating underlying insults. This hypothesis is further supported by the fact that increase in evoked potential latencies cannot be only ascribe to sedation since long-term swallowing disorders [67] and aspiration pneumonia are more frequent in sepsis survivors [68].

Thus, a multimodal assessment of brainstem dysfunction in critical illness is warranted. The undergoing multicenter PRORETRO study (ClinicalTrials.gov: NCT02395861) aims to evaluate a multimodal approach based on neurological examination and neurophysiological tests.

### Mechanisms of brainstem dysfunction

Neuroimaging and neuropathological studies show that the brainstem is prone to vascular, inflammatory, and excitotoxic insults [5]. For instance, sepsis can be associated with impaired autoregulation of cerebral blood flow and microcirculatory dysfunction, which may compromise the brainstem perfusion. Second, a multifocal necrotizing leukoencephalopathy involving the brainstem can be secondary to an intense systemic inflammatory response [69]. Finally, the neuro-inflammatory process can culminate in neuronal apoptosis, which is evidenced in brainstem autonomic nuclei in patients who died from septic shock or in experimental sepsis [5]. Interestingly, it has been shown that apoptosis of autonomic nuclei can induce hypotension in septic rat [70].

Both humoral and neural pathways can induce a neuro-inflammatory process. The former involves the *area postrema* (Fig. 1), which allows the diffusion of circulating inflammatory mediators into the brainstem; the latter involves mainly the vagal nerve, which mediates the transmission of peripheral inflammatory signals to the brainstem [71, 72]. Autonomic brainstem nuclei are regulated by these two pathways, which then play a major role in the control of systemic inflammatory response.

Finally, metabolic processes can be involved. It is well known that electrolyte disturbances but also renal and liver failure impair brainstem responses, as illustrated by centro-pontine myelinolysis or by usefulness of FOUR score in hepatic encephalopathy [73].

### Prognostic value of brainstem dysfunction and therapeutic perspective

The predictive value of the neurological examination findings and neurophysiological responses has been assessed in critically ill patients. There is a proportional relationship between the BRASS value and mortality. Interestingly, absence of a grimacing response associated with preserved oculocephalic responses is the most predictive of mortality [29], suggesting that prediction is better when first based on a combination of signs, and second, a decoupling process between the upper and lower part of the brainstem is involved [29]. The absence of EEG reactivity and of SSEP P14 response and increased P14–N20 SSEP latencies are associated with increased mortality [34, 42, 43]. Impaired HR variability and decreased sympathetic control are associated with mortality and organ failure [74].

There are arguments for a relationship between delirium and brainstem dysfunction. The drugs currently used for treating delirium are involving brainstem receptors. Thus, neuroleptics are antagonists of the dopamine D2 and serotoninergic 5HT2A receptors that are prevalent in the brainstem [75]. Dexmedetomidine is a selective agonist of alpha-2 receptor, notably at the level of the LC [76]. The role of the brainstem in patients with delirium is supported by these pharmacological data and further supported by neuropathological findings that demonstrate hypoxic and ischemic insults of the pons in delirious patients [77]. Absent oculocephalic responses and delayed middle-latency BAEP have been associated with delayed awakening or delirium after sedation discontinuation [34]. In neuroanatomical point of view, it is likely that cessation of the oculocephalic response reflects a dysfunction of the ARAS while cessation of the cough reflex reflects a dysfunction of the cardiovascular and respiratory autonomic nuclei. Finally, if conceivable, we do not know to what extent brainstem dysfunction contributes to long-term post-ICU mortality and functional disability.

Another contributing factor of the brainstem dysfunction in critical illness course might be the impaired sympatho-vagal control of the inflammatory response. The vagus nerve first senses and modulates peripheral inflammation, constituting the so-called cholinergic reflex [78]; second, it senses the microbiota metabolites, being a major component of the gut-brain axis [79] (Table 2). The adrenergic system controls the immune system, with alpha and beta-1 receptors being pro-inflammatory and beta-2 receptors anti-inflammatory [80]. It is therefore conceivable that a brainstem-related neuro-immune impairment can contribute to infection, organ failure, or death by facilitating a maladapted immune response. The modulation of the cholinergic reflex by α7nAChR agonists and by vagal nerve stimulation has been proposed in sepsis and critical illness to improve peripheral immune response and reduce organ dysfunction [81]. In addition to its peripheral immune effects, cholinergic modulation and vagal stimulation can promote anti-inflammatory microglial polarization [82]. However, we shall remind that rivastigmine, a cholinesterase inhibitor, is deleterious in critically ill patients. Vagal nerve stimulation is also proposed in refractory status epilepticus [83] and consciousness disorders [22], suggesting its potential but not yet demonstrated effect in critical illness-related encephalopathy. Beta-blockers reduce the mortality in cardiac diseases by attenuating the deleterious effects of sympathetic hyperactivation and increasing the vagal tone [84]. In sepsis, beta-blockers improve HR control, reduce systemic inflammation, and decrease mortality, acknowledging that their routine use is not yet warranted [85, 86].

## Conclusion

Brainstem dysfunction can present with central sensory and motor deficits, cranial nerve palsies and abnormal brainstem reflexes, disorders of consciousness, respiratory failure, and dysautonomia. Clinical examination is essential for detecting a brainstem dysfunction that may be supported by neuroimaging, electrophysiological, autonomic, and respiratory assessments. Brainstem dysfunction mainly results from secondary insult and might contribute to critical illness-related mortality, organ dysfunction, immune dysregulation, delayed awakening, and delirium. The assessment of the brainstem should then be included in the routine neuromonitoring of critically ill patients.

## Data Availability

Not applicable

## Abbreviations

ADEM: Acute disseminated encephalomyelitis
ANS: Autonomic nervous system
ARAS: Ascending reticular activating system
BAEP: Brainstem auditory evoked potentials
BP: Blood pressure
BRASS: Brainstem Reflexes Assessment Sedation Scale
CAM-ICU: Confusion Assessment Method for the ICU
CRS-R: Coma Recovery Scale-Revised
CSF: Cerebrospinal fluid
ECG: Electrocardiogram
EEG: Electroencephalogram
EMG: Electromyogram
FOUR: Full Outline of UnResponsiveness
GABA: Gamma-aminobutyric acid
GCS: Glasgow Coma Scale
GRpF: Parafacial respiratory group
HF: High frequency
HR: Heart rate
ICCT: Intracranial conduction time
ICDSC: Intensive care delirium screening checklist
ICU: Intensive care unit
IPCT: Intrapontine conduction time
IPL: Inter-peak latencies
LC: Locus coeruleus
LF: Low frequency
MCS: Minimally conscious state
MRI: Magnetic resonance imaging
MS: Multiple sclerosis
MSA: Multiple systematrophy
NMDA: *N*-methyl-d-aspartate
NMO: Neuromyelitis optica
P0.1: Occlusion pressure measurement
PCR: Polymerase chain reaction
PreBotC: Pre-Botzinger complex
PTSD: Post-traumatic stress disorder
RASS: Richmond Agitation Sedation Scale
RE: Rhombencephalitis
RR: Respiratory rate
SSEP: Somatosensory evoked potentials
TDM: Tomodensitometry
Ti: Inspiration duration
Vt: Tidal volume
